# A Reference Hand-book of the Medical Sciences

**Published:** 1887-12

**Authors:** 


A REFERENCE HAND-BOOK OF THE MEDICAL SCIENCES. By various writers. Illustrated by chromo lithographs and fine wood engravings. Edited by Albert H. Buck, M. D. Volume V. New York: William Wood & Co. 1887.

This volume does not fall short of the four preceding publications in the practical nature of the subjects included and in their elaborate discussion by competent authors. While it is entirely impracticable to give even a general idea of the contents, the attention of readers may be drawn to a few of the articles which may serve as specimens of the character of the work. It may be noted at the outset that the paper on periodicity of disease, by Rudolph Matas, of New Orleans, and that on the relations of the portal system to pathology and therapeutics, by F. Peyre Porcher, of Charleston, are the only contributions from any writers south of Baltimore and Washington City.

The attention of the editor and publishers may also be drawn to the omission in the list of contributors to this volume of the name of Thomas M. Markoe, of New York, who furnishes an exhaustive paper on necrosis.

A well illustrated article on plastic surgery, by Joseph Ransohoff, is a timely offering in this department. The following contributions are among those entitled to special consideration: Injuries and diseases of the pancreas, by N. Senn; diseases and new growths of the ovary, by H. C. Coe; injuries and diseases of patella, by George R. Fowler; ozena (simple), by E. C. Morgan; nose, by J. Nolan Mackenzie; pelvic cellulitis, by W. W. Jaggard; nerve tissue, by Henry Koplik; neurasthenia, by J. J. Putnam; anomalies of muscles, by F. J. Shepherd; optometry, by John Green, with good illustrations; ophthalmoscope, by W. O. Moore, well illustrated and a very fine colored plate of the normal fundus oculi; medical otology, by Albert H. Buck, brief, but comprehensive; opium habit, by C. P. Bancroft, which meets an urgent demand; nutrition, by R. H. Chittenden; pneumonia in children, by W. P. Northrop; personal nomenclature, by J. F. Baldwin, suited to the times and worthy of special attention; œsophagus and its diseases, by F. A. Manning, of great practical importance; paralysis, by Henry W. Berg; pelvis, by Frank Baker; congenital malformations of the penis, by Abner Post; inflammation of the pericardium, by Edward Tunis Bruen; primary perineorrhaphy, by J. Johnson Alloway; acute and chronic peritonitis, by George Ross; tumors of the pharynx, by D. Bryson Delavan; photo-micosgraphy, by George M. Sternberg; poisonous insects, with illustrations, by Charles Valentine Riley; post-mortem examinations, by W. W. Gannett, and ending with Pott's disease, by Edward H. Bradford.

